# Toutouwai display positive judgement bias when tested in the wild

**DOI:** 10.1007/s10071-025-02004-6

**Published:** 2025-10-29

**Authors:** Rachael C. Shaw, Hanne Løvlie

**Affiliations:** 1 School of Biological Sciences, Victoria University of Wellington, Wellington, 6012 New Zealand; 2 IFM Biology, Department of Physics, Chemistry and Biology, Linköping University, Linköping, 58381 Sweden

**Keywords:** Cognitive bias, Individual differences, Optimism, Toutouwai, *Petroica longipes*

## Abstract

Cognition, how individuals perceive, store, process and respond to information, influences decision-making. However, biases in cognitive processes can distort behavioural outcomes. Judgement bias occurs when individuals infer information to be more positive, or negative than it is. For captive animals, judgement bias is typically measured by testing behavioural responses to ambiguous cues that are intermediate to cues with learned valence (positive and negative). However, these tests have rarely been used in the wild. We therefore examined if a behavioural test of judgement bias commonly used in captive animals could be adapted to test a wild bird, the toutouwai (North Island robin, *Petroica longipes*). Toutouwai were faster to approach ambiguous cues that were more similar to a learned positive cue, compared to a learned negative cue. This positive bias mirrors behavioural response patterns observed across a range of species in captivity, including other birds. Responses toward ambiguous cues were repeatable over a short timespan, indicating there was consistent individual variation among our test birds in their degree of bias when judging ambiguous cues. Males tended to approach cues more quickly than females. Our results demonstrate that judgement bias can be tested for in situ in wild birds. However, as is typical of many island species, toutouwai are bold and fearless, which facilitated their participation in our experiment. Therefore, to enable research examining the ecological correlates of judgement biases in nature, we must develop tests of cognitive biases that can be used with a wider range of species in the wild.

**Supplementary Information:**

The online version contains supplementary material available at 10.1007/s10071-025-02004-6.

## Introduction

Cognition (i.e., how individuals perceive, process, store and act on environmental information, Shettleworth 2010), has a long research history, particularly in a comparative framework. More recently, within-species variation in cognitive performance has been associated with differential fitness outcomes in the wild (Cole et al. 2012; Madden et al. 2018; Shaw et al. 2019; Welklin et al. 2024). However, cognition can contain biases and individuals can show consistent deviation from an accurate perception, or judgement, of information (reviewed in Fawcett et al. 2014; Paul et al. 2005; Sharot 2011). One such set of biases is for animals to behave as though conditions are better, or worse, than they actually are, producing ‘optimistic’ or ‘pessimistic’ judgement biases, respectively (Paul et al. 2005; Fawcett et al. 2014). Understanding the nature of such biases is of broad relevance to all behavioural research, as they can distort expected responses during cognitive and behavioural assays, but may also explain observed variation. As research on quantifying individual differences in the cognitive performance of wild animals continues to accumulate, developing our understanding of whether and how cognitive biases are expressed in the wild will become increasingly relevant.

Decision-making under ambiguity can be used to assess judgement biases (Nygren et al. 1996; Paul et al. 2005; Sharot et al. 2012). In humans, for example, judgement bias can be measured by asking individuals to verbally estimate their own future success (Nygren et al. 1996; Sharot 2011; Sharot et al. 2012). For animals, behavioural tests have been developed to measure judgement biases. The general approach is to observe how individuals respond to novel, ambiguous cues that are intermediate between cues with learned positive and negative valence (i.e., learned cues that an animal has been trained to associate with a reward or with no reward/punishment; see figure of general test procedure in Lagisz et al. 2020). This paradigm was first established in a seminal study by Harding and colleagues (2004), which measured the behavioural responses of rats (*Rattus norvegicus*) to ambiguous acoustic tones that were intermediate between a learned tone that signalled white noise (negative valence), and a learned tone that signalled a food reward (positive valence). Rats housed in poorer conditions responded pessimistically to the novel, intermediate, ambiguous cue, while rats in normal housing conditions behaved optimistically to the same ambiguous cue (Harding et al. 2004). ‘Optimism’ and ‘pessimism’ were inferred according to whether responses towards ambiguous cues were more similar to the cue with positive valence, or the cue with negative valence, respectively (Mendl et al. 2009; Paul et al. 2005; such terms are also used to describe biased judgements in humans, e.g., see Sharot et al. 2012). Judgement bias tests have since been developed to use cues based on perceptual senses other than hearing. In birds, visual cues are often used as a substi2018btute for tones. For example, shades of grey cues have been used in starlings (*Sturnus vulgaris*, Bateson and Matheson 2007), domestic fowl (*Gallus g. domesticus*, Zidar et al. 2018b), and red junglefowl (*Gallus gallus*, Garnham et al. 2019, 2022; Sorato et al. 2018). Tests based on the ambiguous cue paradigm have now been used in a range of species, which have displayed judgement bias in spatial, visual, auditory, tactile and olfactory based versions of the test, confirming the widespread existence of judgement bias (reviewed in Lagisz et al. 2020).

There is ongoing debate as to the nature of the inner states (including affective and motivational states) and cognitive abilities that give rise to biased responses towards ambiguous cues in non-human animals (Mendl et al. 2010; Nematipour et al. 2022). Nevertheless, judgement bias tests have been used to infer individuals’ affective states (feelings, mood) in an animal welfare context (reviewed in Lagisz et al. 2020). The judgement bias test is an important and rare tool for assessing positive animal welfare (Lagisz et al. 2020). As a result, most studies on judgement bias are done on domesticated species and in captivity (e.g., sheep, *Ovis aries*: Doyle et al. 2010; domestic fowl: Zidar et al. 2018b). The exploration of judgement biases is also of relevance in fundamental animal behaviour research. For example, judgement bias has been examined in contexts including mate choice (e.g., convict cichlid, *Amatitlania siquia*: Laubu et al. 2019) and reproductive investment (e.g., zebrafish, *Danio rerio*: Espigares et al. 2022). However, few studies have focused on understanding judgement bias in the wild per se (Lagisz et al. 2020). Wild-caught animals have been tested in a captive setting using standard versions of the judgement bias test (e.g., wild starlings, Bateson et al. 2015), and two studies have coopted naturally occurring aversion behaviours (e.g., to predators) to create novel and ecologically relevant attention bias (e.g., Starlings, Brilot et al. 2009) and judgement bias (e.g., Murray cod, *Maccullochella peelii*, Freire and Nicol 2024) tasks for wild-caught, or wild animals. To date, the standard version of a judgement bias test where an animal first learns the valence of a novel negative and a novel positive cue, before being presented with novel, ambiguous cues, has not been attempted in wild animals *in situ* (Lagisz et al. 2020). This is likely because the repeated trials and standardised testing conditions that are required to first train positive and negative cue valence and then test responses to ambiguous cue are logistically challenging to implement in free-living animals (Freire and Nicol 2024). Therefore, our current understanding of judgement biases in non-human animals largely comes from experiments in captivity, where the environment is typically simplified relative to decision-making contexts in nature (Fawcett et al. 2014). However, given that environmental richness can alter decision-making biases (Zidar et al. 2018b; Anderson et al. 2021; Buenhombre et al. 2023), testing cognitive judgement bias in the natural habitats in which animals live is important.

Additionally, quantifying judgement bias in the wild could create opportunities for studying the ecological correlates of individual variation in a cognitive bias in non-human animals. However, such research requires establishing whether behavioural-based tests of judgement bias are feasible in natural populations and can produce responses comparable to similar tests already carried out in captive settings. It would also be valuable to explore whether there are consistent individual differences in judgement biases in natural populations. Achieving the latter requires estimating the repeatability of judgement bias task performances, as a first step to validate the consitency in responses of individuals. Repeatability is a measure of the behavioural variation that is due to consistent differences among individuals within a population – when among individual behavioural variation is high and within individual behavioural variation is low, the behavioural trait has high repeatability (Bell et al. 2009). For future research aimed at addressing the evolutionary causes and consequences of individual variation in judgement biases, quantifying repeatability can set an upper limit for the heritability of judgement bias (Boake 1989). Previous research in captive junglefowl revealed moderate repeatability (however, with large standard errors) in individual responses towards ambiguous cues during a judgement bias task (Sorato et al. 2018), suggesting that there are consistent individual differences in how individuals respond to ambiguity. However, the repeatability of judgement bias task performance remains unknown for natural populations of any species in the wild.

The toutouwai (North Island robin, *Petroica longipes*) is a small insectivorous passerine bird that is endemic to forests in the North Island of New Zealand. Toutouwai are bold and curious, typically lacking fear toward people. As a caching species, toutouwai store invertebrate prey over short time intervals and so a bird will remain motivated for food rewards even when satiated (Vámos and Shaw 2024). Moreover, as an individual will hold and defend the same territory for long durations, which is often their entire lifespan in our population (up to 13 years), individuals can be repeatedly tested (Shaw and Harvey 2020). In the past decade, wild toutouwai have been tested in a range of cognitive and behavioural tasks including associative and reversal learning (Shaw et al. 2015; McCallum and Shaw 2023a), spatial memory (Shaw et al. 2019; Vámos and Shaw 2025), long-term memory (Shaw and Harvey 2020), self-control (McCallum and Shaw 2024) and inhibitory control (Shaw 2017; McCallum and Shaw 2023a). Toutouwai are also one the few species for which we now have evidence that individual variation in cognitive performance is linked to behaviour and reproductive success in the wild (Shaw et al. 2019). Their willingness to participate in a range of cognitive tasks in the wild and motivation for food rewards make toutouwai ideal subjects for implementing the judgement bias paradigm in the wild.

In the current study we investigate whether a cognitive judgement bias task that has been widely implemented in laboratory and captive animals can be adapted for use with free-living wild birds. To achieve this, we tested whether toutouwai show evidence of judgement bias in their behavioural responses to novel, ambiguous visual cues. We first used a black/white colour discrimination task to train each bird to learn the valence of these two reference cues; one was rewarded, and one was unrewarded. Once a bird had learned the discrimination task, we then evaluated cognitive judgement bias by measuring their latency to approach an individually presented visual cue. The cue was either already known (i.e., black, or white) or one of three intermediate, novel and ambiguous grey scale cues (see methods for details). We have previously found that female toutouwai have more accurate short term spatial memory performance than males (Vámos and Shaw 2025) and so we also explored whether sex differences existed in the approach responses in our judgement bias task. In other species, judgement bias studies including both sexes have produced mixed results (e.g., Berlinghieri et al. 2024; Sorato et al. 2018). Finally, to understand the extent to which individuals are consistent in their behavioural responses during the judgement bias test, we also evaluated whether individual performances in the task were repeatable over short durations. We have previously shown that toutouwai have low to moderate repeatability over short (days or weeks) and long (1 year) durations in a range of behavioural (McCallum and Shaw 2023b; Vámos and Shaw 2024) and cognitive task measures (Shaw et al. 2019; McCallum and Shaw 2023a). We therefore predicted that birds would show temporal consistency in their responses towards ambiguous cues in our judgement bias task when tested over a short timespan (two days).

## Methods

### Study population

The birds that participated in this research belonged to our long-term study population of wild toutouwai at Zealandia Sanctuary Te Māra a Tane in Wellington, New Zealand. Our study population has been continuously monitored since 2014 and the birds inhabit an approximately 25 ha area of mixed regenerating native forest and introduced pine forest inside Zealandia. The median breeding territory size in our population is 0.36 ha, range = 0.13–0.83 ha (Shaw et al. 2019). However, during the autumn and winter a breeding pair will divide their territory and avoid each other when foraging (Menzies and Burns 2010). This territorial beahviour allows birds to be tested individually in Austral winter months. All birds had previously participated in behavioural-based cognition experiments for mealworm (*Tenebrio molitor*) rewards and were habituated to the presence of an experimenter on their territory (Shaw et al. 2015, 2019). Birds were individually banded with a unique combination of 3 plastic-coloured bands and one metal band (following guidelines of the New Zealand National Bird Banding Scheme).

### General testing procedures

Testing took place during the Austral winter between 29/07/2019 and 30/08/2019, when birds defend a winter territory (Menzies and Burns 2010). Focal birds were chosen based on whether they had a location on their winter territory that was sufficiently flat for testing and at least 5 m from the territory boundary, which can be readily identified by behavioural displays (Shaw et al. 2019). We began our testing with 24 adult individuals (11 males and 13 females). Ten males and nine females completed the entire experiment (mean age 4.2 years, range 1.5–11.5 years, Table S1). For each subject, we chose a testing location as close to the centre of their territory as possible to minimise interference from neighbouring conspecifics. If a conspecific appeared, we suspended testing until it had left the vicinity, or been chased away by the subject (Shaw et al. 2019) Freshly killed mealworms were used as rewards throughout testing. Birds started and ended a testing session by stepping onto a familiar platform for a mealworm reward to establish whether they were food motivated. Birds were familiar with this procedure from earlier experiments and all birds in the current study readily approached and always took these mealworms. All trials were filmed on an iPad to allow for behaviours to be scored from videos. During all trials, the experimenter positioned themselves approximately 1.5 m away in a central position behind the apparatus. The experimenter sat or stood, depending on the terrain, and observed the movement of the bird on the iPad screen, to avoid looking directly at the focal bird during trials.

### Discrimination task

**Fig. 1:**
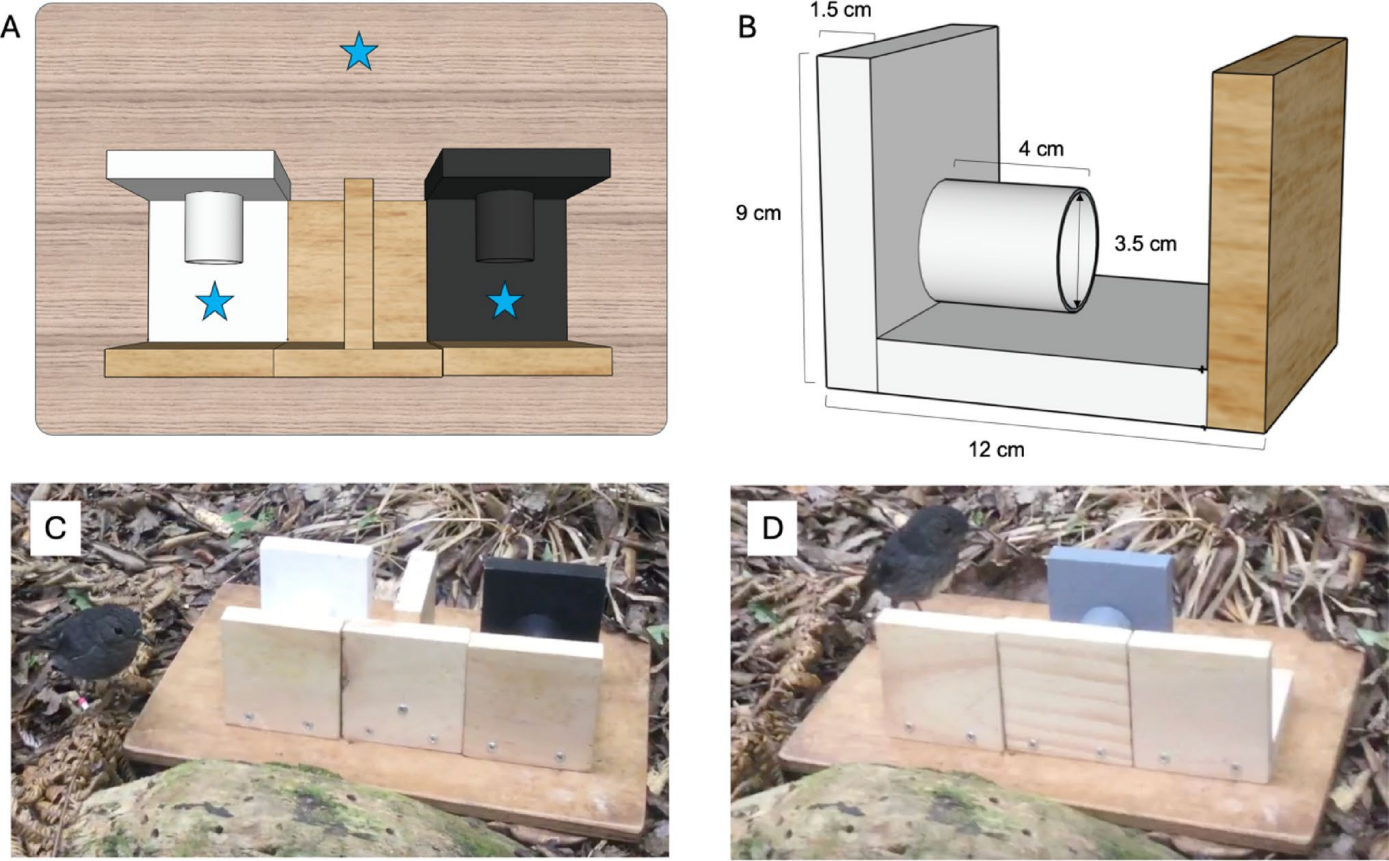
The apparatuses used for the discrimination training and the judgement bias test in wild toutouwai. (A) An aerial view of the discrimination training apparatus on the wooden testing platform (platform dimensions, H × D × W: 1.5 × 25 × 35 cm). The blue stars denote the location of the mealworm rewards during an initial habituation trial. (B) A schematic of the white cue, showing the dimensions. If the cue was rewarded, the mealworm was placed inside the tube, approximately 1 cm from the entrance. (C) The apparatus during a discrimination training trial, with the black and white cues separated by a wooden barrier. One mealworm was placed inside the tube of the rewarded colour, so that it was only visible when the bird looked into the tube opening (as the bird is shown here looking into the tube behind the white cue). (D) An example of a novel ambiguous cue presentation (the Middle cue) during the judgement bias test. Ambiguous cues were unrewarded

For the discrimination task, we presented each focal bird with two cues (black, and white) placed on a wooden testing platform (Fig. 1A). Each cue consisted of a painted square wooden barrier concealing an opaque tube painted the same colour (Fig. 1B). The tube was mounted on a wooden base painted to match the tube and barrier. Opposite the tube’s opening there was an unpainted wooden barrier, which created a 5 cm gap that the bird needed to place its head into to be able to reach the reward inside the tube (Fig. 1B). A wooden wall separating the black and white cues ensured that a bird could only see behind one cue barrier at a time (Fig. 1A and C). The left/right location of the black cue and white cue remained consistent across all trials for an individual. We counterbalanced cue location among birds, so that for the 24 individuals that began the discrimination task, white was always on the right side of the testing platform for 12 of the birds and on the left side of the platform for the other 12 birds.

Before the discrimination task began, we first habituated a bird to the apparatus and gave it an opportunity to learn that the apparatus could contain mealworms. Habituation had two steps. In the first step we placed one mealworm ca. 10 cm in front of the apparatus in the centre of the platform, and a mealworm behind each cue barrier, just outside the tube entrance (Fig. 1A). We allowed the birds up to five minutes to take all three mealworms. If a bird timed out, we removed any remaining mealworms for ca. 1 min, then re-baited all three locations. Once a bird had taken all three mealworms within 5 min in two consecutive trials, they moved to the next habituation step. In the second step, we placed one mealworm inside each tube and one mealworm front and centre on the board, ca. 10 cm from the apparatus. This second habituation step ensured that a bird had explored the entire apparatus and learned that the tubes could contain mealworms. To pass the second habituation step, a bird had to take all three mealworms in three consecutive trials. During the habituation stage, we gave each bird up to four trials per testing session; birds took approximately 6 trials (range 5–10 trials) to pass both steps (see Table S1). After a bird had passed the second habituation step, we immediately began discrimination training on the same day by giving the bird discrimination trials (methods described below) until it had received a maximum of 15 mealworms for that testing session (across both habituation and discrimination trials).

We initially attempted habituation with 11 males and 13 females. However, one male never learned to reliably look inside the tubes during 11 trials of the second habituation step, one female never passed the first habituation step, and 3 females were continuously displaced or interrupted by their mates in all of the testing locations that we attempted on their winter territories. These five individuals were therefore excluded from discrimination training.

For the discrimination training, we assigned the rewarded/unrewarded cues to each bird based on the individual’s most common first cue selection during habituation. To ensure that birds did not reach the discrimination learning criterion simply because they were either side-biasing or choosing their most preferred colour option, whichever cue the bird had most frequently visited first in the habituation phase became the unrewarded cue (i.e., it therefore needed to learn that its non-preferred side/colour was the rewarded one, see Table S1 for rewarded colour and side for each bird). White became the rewarded cue for 8 birds (5 female, 3 male) and black was the rewarded cue for 11 birds (7 male, 4 female).

For discrimination training, we gave birds up to 15 trials per testing session. If required, a bird received testing sessions on consecutive days until it had completed the discrimination training (except for six birds, where poor weather prevented testing on subsequent days). When placing the mealworm reward inside the apparatus, the experimenter held both cues, turned their back towards the bird, and baited the rewarded cue out of view of the bird. When the bird was within 5 m of the testing platform the experimenter began a trial by placing the apparatus down on the platform and stepping back approximately 1.5 m (and sitting down if feasible). During a trial, we gave the bird up to 3 min to retrieve the worm from inside the rewarded tube. If it first approached, or stood, on the non-rewarded side of the apparatus in a position that potentially gave it a view inside the tube (e.g., Fig. 1C), the trial was deemed an incorrect response, and the apparatus was removed. If it first approached the rewarded colour side and retrieved the worm, it was considered a correct response. We ended a trial either once the worm was retrieved, the bird had made an incorrect choice, or after 3 min had elapsed, whichever occurred first. A time out at 3 min only occurred in one trial, due to a female preening. The two stimuli were removed between trials and there was an intertrial interval of approximately 1 min between each trial to record the outcome and reset the apparatus. We considered a bird to have learned the discrimination when it had made 10/12 consecutive correct choices, which could occur across two successive testing sessions on consecutive days.

### Measuring judgement bias

After completing the discrimination task, each bird received two test sessions on consecutive days in a judgement bias test. Immediately prior to a test session, we gave a bird three “refresher trials” (sensu Zidar et al. 2018b, 2019) of the discrimination task, to ensure it continued to prefer the learned, rewarded cue. Across all trials for all birds, the learned, rewarded cue was visited first in all but two of the refresher trials. For a test trial, we presented the bird with a single cue in the middle of the platform (Fig. 1D). In addition to the familiar black and white cues, we used three novel, unrewarded ambiguous grey scale cues (‘light grey’ – 75% white and 25% black, ‘mid grey’ 50% white and 50% black, and ‘dark grey’ – 25% white and 75% black). These ambiguous cues were therefore Near positive, Mid, and Near negative, with the light grey cue being Near positive for birds trained on white as their rewarded Positive cue, and the dark grey cue being Near positive for birds trained on black as their rewarded Positive cue. During judgement bias test trials, only the familiar, rewarded cue contained food (based on the colour of the cue each individual bird was trained on). A session contained 12 test trials in total, with two presentations of each ambiguous cue and three presentations of the familiar Positive (rewarded) and Negative (unrewarded) cues. Within a test session, the order was balanced so that each ambiguous cue was presented once after the Positive (familiar rewarded) cue and once after the Negative (familiar unrewarded) cue. The overall trial order was counterbalanced among birds to control for the possibility that the order in which the ambiguous cues were experienced within a session affected approach latencies. The order of cue presentations used within each session is provided in Table S2. A trial ended once a bird had approached the test apparatus (see below). We then removed the cue from the testing platform and placed it into an opaque bag. To mimic the intertrial duration and procedures used in the discrimination training, we turned our back to the bird to record the trial outcome and select the next cue from this bag.

To quantify judgement bias, we measured the latency in seconds for individuals to approach each cue. These latencies were coded from videos by someone who was initially blind to the cue that had been learned by each bird and the identity and sex of each bird. Videos were played and a stopwatch was used to measure approach latency. Latency to approach was measured from when the experimenter stopped touching the apparatus and stepped backwards (which only happened after the focal bird was within 5 m of the testing platform, see above). In trials where the bird was in a position where the apparatus was not within its view when this happened (e.g. because the bird was downslope from the apparatus and below a bank, or behind a tree or foliage), the trial began as soon as the experimenter stated on the video audio that a bird had moved to a position where it could now view the apparatus. The timer was stopped when the bird’s feet first contacted the apparatus (either the cue itself, or the testing platform) by either stepping onto it, or by flying and landing onto it. We chose to use approach latencies rather than proportion of trials in which birds approached as the measure for our task, as the toutouwai showed a remarkably high probablilty of approaching all cues (see results) and using approach latency is recommended best practice by a meta-analysis (Lagisz et al. 2020).

### Statistical analyses

All analyses were run in R version 4.4.1 (2024-06-14), with alpha set at 0.05. To evaluate toutouwai behaviour during the judgement bias test, we modelled how Cue type (Positive, Near positive, Mid, Near Negative, Negative, coded as either continous or categorical, see below) and Sex (a variable with two levels: Male and Female) influenced the latency to approach a cue (s). In these analyses, we excluded trials in which a bird was preening before approaching the test platform (N_trials_ = 3), was interrupted by, or chased a conspecific from their territory (N_trials_ = 2), or did not approach the cue within 5 min (N_trials_ = 7; 5 trials towards a Negative cue, 1 trial towards a Near negative cue, and 1 trial towards a Mid cue). Our latency to approach models therefore included 444 samples in total.

A mixed modelling approach that incorporates all necessary hierarchical levels in the random effect term is the recommended best practice for the statistical evaluation of cognitive judgement bias tests (Gygax 2014). As our latency data was right skewed, we modelled data using a Gamma distribution with a a log link function (Berlinghieri et al. 2024). We specified Generalized Linear Mixed Models with a gamma distribution and logarithmic link, using the ‘glmmTMB’ package (Brooks et al. 2017). Model assumptions were checked using the ‘DHARMa’ package (Hartig 2019). To account for the structure of our cognitive judgement bias test, where each subject had 2 test sessions with 12 trials per session, we specified a random effects structure with Trial number nested within Session, and Session nested within Individual identity. This structure allows for the assessment of the response to cue types while accounting for variability between individuals, between sessions for each individual and between trials within each session.

Following the recommendations of Gygax for quantitiatively comparing the values of each cue type (2014), in our models we coded Cue type as a discrete continuous variable, where 1 represented the learned rewarded Positive cue, 2 the novel ambiguous Near positive cue, 3 the novel ambiguous Mid cue, 4 the novel ambiguous Near negative cue and 5 the Negative unrewarded cue (see supplementary data). We initially included an interaction between Sex and Cue type to examine whether the sexes had differing slopes for their responses to the cues. The interaction with Sex was not significant and so was dropped to properly evaluate the main effects (Gygax 2014). Therefore, the final model that we used for inference included Cue type and Sex as fixed effects. This model was also re-run with Cue type specified as categorical, to test for differences in responses to specific cues, allowing for post hoc Tukey test comparisons of the cue responses, using the ‘multcomp’ package (Hothorn et al. 2008).

Finally, to examine the individual consistency in behavioural responses toward ambiguous cues during the judgement bias test, we calculated the repeatability of the latency to approach ambiguous cues (i.e., the latency to approach in trials with the Near positive cue, Near Negative cue and Mid cue, *N* = 223 trials). We used the ‘rptR’ package (Stoffel et al. 2017) and fit two Linear Mixed Model-based repeatability models. In both models we used the log transformed latency to approach the cue as the response, with Individual identity included as a random factor. The first model estimated adjusted repeatability whilst accounting for learning effects over repeated trials, by including cumulative Trial number (1–24) as a fixed factor. The second model estimated unadjusted repeatability by only including the Individual identity random factor. We calculated the bootstrapped 95% confidence intervals for each repeatability estimate.

## Results

**Fig. 2:**
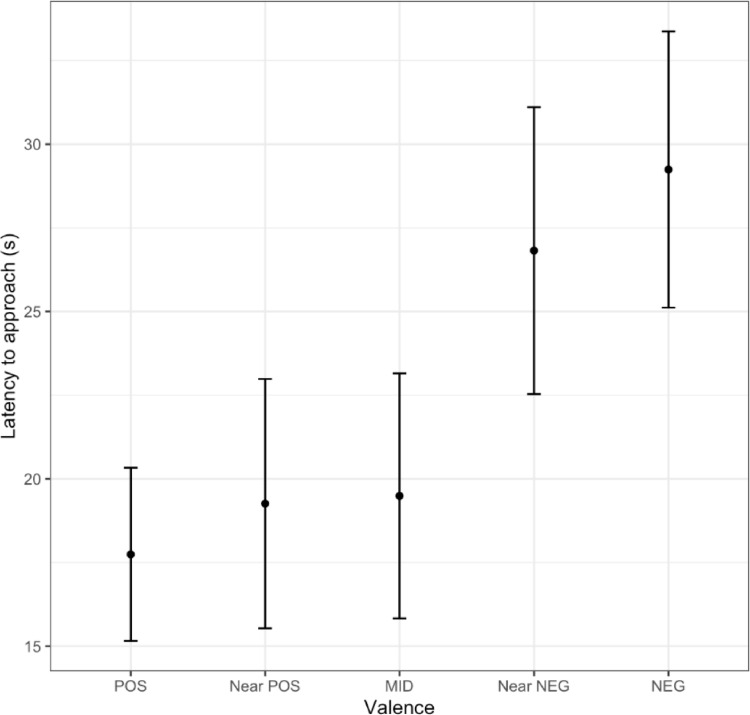
Behavioural responses of wild toutouwai during a judgement bias test. We measured latency (s) to approach each cue type (POS = Positive, Near POS = Near positive, MID = Middle, Near NEG = Near negative, NEG = Negative). The black, filled points with error bars display the mean approach latency and standard error for each cue type. Supplementary material figure S1 shows these means together with all individual latency measures

All 19 birds learned the initial colour discrimination. On average, birds took 26.6 ± 3.2 (mean ± S.E.) trials to reach the pre-set learning criterion of 10 of 12 correct consecutive choices. Furthermore, in the judgement bias test, birds were faster to approach the Positive than the Negative cue (Fig. 2; Table 1). Collectively, birds approached 97.8% of the ambiguous cues in the judgement bias test. Out of the 228 ambiguous cue presentations there were only five trials in which a bird did not approach the cue – in three of these cases this was due to interruption by a con- or heterospecific.

In the model of approach latency with Cue type coded as a continuous variable, there was an effect of Cue type (estimate ± S.E. = 0.107 ± 0.027, *z* value = 3.996, *p* < 0.0001) suggesting that approach latency increased as cue types moved away from positive and toward negative valence (Fig. 2). There was also a tendency for males to approach faster than females across all cues (specifying female as reference value, the male estimate ± S.E. = −0.651 ± 0.334, *z* value = −1.951, *p* = 0.051). In the model with Cue type as a categorical factor, there was again an effect of Cue type on latency to approach, and a tendency for males to approach faster (Table 1). Tukey Post-hoc contrasts (Table S3) revealed that compared to the Positive cue, birds were slower to approach the Negative cue (estimate ± S.E. = 0.395 ± 0.115, *z* value = 3.432, *p* = 0.005; Fig. 2). Birds also took longer to approach the Negative cue than the Near positive cue (estimate ± S.E. = 0.379 ± 0.129, *z* value = 2.935, *p* = 0.028), or the Middle cue (estimate ± S.E. = 0.372 ± 0.129, *z* value = 2.893, *p* = 0.031; Fig. 2). Finally, toutouwai showed moderate adjusted repeatability (controlling for Trial number) in their approach latencies during trials with the three ambiguous cues (*R* ± S.E. = 0.502 ± 0.091, 95% C.I. = 0.306–0.644), as well as moderate unadjusted repeatability (*R* ± S.E. = 0.503 ± 0.095, 95% C.I. = 0.310–0.658).

**Table 1:** Parameter estimates and significance tests for generalized linear mixed model of Toutouwai approach latency in a judgement bias test. Model was run with gamma distribution and logarithmic link, with Cue type specified as a categorical factor. To account for the structure of the data, Trial: Session: Identity was specified as a nested random factor. Estimates and test statistics are shown in reference to the Positive cue (Cue 1) for Cue type, and to Female for Sex

	Estimate	S.E.	z value	Pr(>|z|)
(Intercept)	2.698	0.252	10.690	< 2e-16
Near positive (Cue type 2)	0.016	0.128	0.126	0.900
Middle (Cue type 3)	0.023	0.127	0.184	0.854
Near negative (Cue type 4)	0.328	0.128	2.566	0.010
Negative (Cue type 5)	0.395	0.115	3.432	0.001
Sex (Male)	−0.650	0.334	−1.948	0.051

## Discussion

Here we successfully tested a wild bird, the toutouwai, in situ using a behavioural test of judgement bias that was originally developed for domesticated and captive animals. The birds’ behavioural responses toward novel, ambiguous visual cues suggested that toutouwai have a judgement bias. As a group they showed an extremely high probability of approaching all cues during the test, including both the learned cues and novel ambiguous cues. Moreover, the approach latencies for ambiguous cues (Near positive cue, and Mid cue) resembled individuals’ responses toward the cue with the trained, positive valence. In captive animal studies, such responses are interpreted as a positive judgement bias (Paul et al. 2005; Mendl et al. 2009). Furthermore, evidence for moderate repeatability in the behavioural response towards the three novel, ambiguous cues suggests that individuals consistently differed in their judgement bias responses. We also observed a tendency for males to approach cues faster than females. Below, we first discuss potential sources of bias in our data, the validation of our test and consistency in responses, before discussing how our observations correspond to other studies and exploring why individual toutouwai may act in a biased, optimistic manner.

Animal cognition and behaviour research can lack transparency in terms of the subject attrition rate (Webster and Rutz 2020). However, the exclusion of individuals prior to the final dataset can bias statistical analyses and the interpretation of observed patterns (Roelofs et al. 2016). For example, in the red junglefowl, personality can correlate with cognitive performance (Zidar et al. 2018a) and optimistic judgement bias with social rank (Garnham et al. 2019). Hence the loss of, or overrepresentation of, certain behavioural types (e.g., bold or more dominant individuals) could bias our interpretation of observed patterns (Webster and Rutz 2020). Here we set out to test 24 (11 males, 13 females) birds and 19 of these (10 males, 9 females) passed all stages of training and testing. Thus, data from 21% (5 individuals) was excluded from our final analyses (9% of males, 31% of females). Potentially, this level of subject attrition could have biased our results in a systematic manner. However, the majority of the subjects that did not complete testing were excluded due to an external factor (i.e., three females were chased away during testing by their male partner), rather than an intrinsic lack of motivation or aversion to the task. Disruption due to external factors accounts for why most cognitive testing is done on isolated individuals, both in the wild and in captivity. Moreover, aiming for larger sample sizes in behavioural tests can help minimise the likelihood and impact of systematic biases in the final collected dataset.

For the 19 individuals that completed our experiment, we observed responses suggesting a strong positive judgement bias. In 97.8% of all presentations of an ambiguous cue, the individual approached the cue. Latencies to approach the Near positive and Mid ambiguous cues were also closer to the responses individuals had towards the cue with positive valence (vs. the cue with negative valence), suggesting that wild toutouwai had a positive judgement bias in their interpretation of the novel, ambiguous cues in our test. We observed a monotonic response curve for approach latency (i.e., the mean response latency increased across cue types in the same order as cues types changed with respect to the learned cue), as has been documented in other studies on judgement bias (e.g., Harding et al. 2004; Hintze et al. 2018; Neuhauser et al. 2023; Zidar et al. 2018b). These indications that toutouwai were interpreting ambiguous cues in reference to the learned cue with the positive valence partially validates our task as a test of judgement bias (Gygax 2014). However, as a further validation, future research on judgement bias in wild toutouwai should ideally include an intervention aimed at altering subjects’ affective states. Demonstrating that responses to ambiguous cues shift in predictable ways (e.g., toward the positive or negative valence, depending on the nature of the intervention), would further support the possibility that toutouwai have an underlying positive judgement bias when evaluating ambiguous cues.

Across two days of testing we observed moderately strong repeatability in individuals’ behavioural responses towards the ambiguous cues. This behavioural consistency mirrors reponses seen in captive junglefowl populations that were tested in a similar judgement bias task (Sorato et al. 2018). This may suggest that there are consistent individual differences in the degree to which toutouwai have positive judgement bias (e.g., some toutouwai may be consistently more likely to make ‘optimistic’ judgements). This finding opens up the possibility for future research examining the ecological causes and consequences of individual differences in judgement bias in the wild. Moreover, as repeatability can set the upper limit for heritability (Boake 1989), our repeatability estimates could suggest that the heritability of judgement bias variation in toutouwai is similar to the heritability estimates found in a previous study in captivity, which were moderate (although with large standard errors, Sorato et al. 2018). However, to further explore these possibilities, we must first estimate the repeatability of toutouwai responses in a judgement bias task across much longer timeframes than were measured in the current study. An alternative explanation for the moderately strong repeatability we report here is that it reflects the consistency in an individual’s testing environment across two days, as well as capturing the subtle variations in testing environments between individuals, given the heterogeneous nature of toutoutwai territories. For example, the structure of the forest understory at the testing site may have led to some birds having more consistent starting locations or preferred approach pathways in our task, which could influence approach latencies. To overcome this, future research could implement a standardised ‘starting perch’ where birds are trained to wait between trials (e.g. McCallum and Shaw 2024).

In our judgement bias test we found a tendency for males to approach all cues faster than females. In previous judgement bias research, both sexes were not always included, or sex effects were not directly tested. However, in sticklebacks (*Gasterosteus aculeatus*), males were more ‘optimistic’ than females (Berlinghieri et al. 2024), while in a large sample of young red junglefowl where both sexes were tested in a judgement bias test, there were no sex-differences observed in judgement bias (Sorato et al. 2018). Since underlying mechanisms of ‘optimism’ often are linked to variation in the dopaminergic systems (e.g., red junglefowl, Boddington et al. 2020; humans, Sharot et al. 2012; bumble bees, Solvi et al. 2016; domestic fowl, Zidar et al. 2018b), which are systems shared among both sexes, sex differences may not necessarily be expected (but see e.g., Neville et al. 2020 for a review of other potential underlying mechanisms). However, male toutouwai are socially dominant to females (Burns and Steer 2006), thus sex differences in traits such as aggression or boldness could account for why males tended to approach cues more rapidly during our judgement bias test. However, given the relatively small sample size in the current study, future research is required to validate the existence of a sex difference in toutouwai judgement bias.

The design of our judgment bias task may have limited applicability for testing a wide range of species in the wild. Judgment bias tasks with a go/go set up, in which an animal chooses between two options, can elicit more responses overall and produce clearer differentiation towards cues (Lagisz et al. 2020). While toutouwai were highly motivated to approach all cues during our go/no-go task, a go/go task could be preferable for testing other species in the wild, as these types of tasks are less likely to be impacted by a lack of motivation (Lecorps et al. 2021). Moreover, judgement bias tests such as the one we used require relatively large amount of pre-training, which makes it harder to do in wild populations. There are currently few examples of judgement bias tests that have been specifically designed to include less training and handling of animals. Using instinctively attractive, or aversive, stimuli provides one such approach to reduce the requirement for extensive pre-training (e.g., for attention bias, Brilot et al. 2009). A recent study on Murray cod, used attractive light versus aversive predator models and intermediate cues, to sample groups of individuals across populations over different environmental conditions (Freire and Nicol 2024). Overall, more research is required to develop tests for cognitive biases that are feasible for use across a wider range of wild species.

The nature of the inner states and cognitive processes that underlie both judgement biases and responses in judgement bias tasks are still debated (Nematipour et al. 2022). In addition to affective states, inner states such as food motivation may affect responses in judgement bias tests. For example, judgement bias tests often entail food rewards (at least for the positive cue) and in a captive setting, individuals may be food restricted prior to testing to increase motivation to participate (e.g., see Box 1 in Lecorps et al. 2021). If both hunger levels and responses to food restriction vary among individuals this could produce variation in test responses among and within test animals, that do not capture decision-making biases. We used a highly attractive food item (mealworm) and measured a bird’s food motivation before and after each session. All of the birds ate the mealworm presented at the end of a test session. This suggests that our birds were highly food motivated throughout testing, with minimal variation in food motivation among individuals. Birds in our population are not supplementarily fed, but do receive mealworms when participating in behavioural experiments, plus a small number (< 10) up to twice per week during breeding season monitoring. The use of mealworms and previous experience of this food in behavioural tests could have translated into the birds being highly motivated to participate in the test.

In our study, we observed a pattern of responses toward ambiguous cues that is commonly interpreted as evidence of positive judgment bias, or ‘optimism’ in animals (Mendl et al. 2009). However, the precise cognitive mechanisms producing the biased responses observed in judgement tasks remains a subject of debate (e.g., Nematipour et al. 2022). For example, reactions toward ambiguous cues could appear positively biased when animals cannot discriminate between learned positive and ambiguous cues, or misrepresent a novel cue as the positive learned cue (Nematipour et al. 2022). However, we consider it unlikely that visual grey scale cues we used for toutouwai are susceptible to these issues. Toutouwai are likely to be extremely sensitive to changes in greyscale, because the darkness of a male’s grey plumage is an indicator of age and territorial dominance (Berggren et al. 2004; Berggren and Low 2006). Theoretically, animals perceiving conflicting contents in the ambiguous cues, or having entirely novel representations of the ambiguous cue may also produce similar response curves as decision-making (Nematipour et al. 2022). Yet, the classic version of the judgement bias tasks, such as the one we use here, cannot easily distinguish these possibilities (Nematipour et al. 2022). It therefore remains plausible that these processes (rather than biased decision-making) may explain the positively biased responses that we and others have observed toward ambiguous cues in the judgement bias task.

Judgement bias is theoretically predicted to be adaptive under natural selection (Fawcett et al. 2014). For example, optimism is predicted when dispersal between environments of varying quality is relatively high, or when predation risk is relatively low (Fawcett et al. 2014). Further, an optimistic judgement bias may not translate into positive over-estimation in all types of situations that an animal faces in the wild, hence an observation in a single context may not reflect the actual consequences of such a bias for an individual (Nygren et al. 1996). In the simplistic testing environment that is typical of captive studies, individuals are removed from the heterogenic and complex environment under which such biases have been selected for (Fawcett et al. 2014). Our observation of an optimistic judgement bias in a wild species confirms that such biases are not only an artefact of a simplistic set up observed in captivity. Both humans (Sharot 2011) and captive animals repeatedly show an overall optimistic bias, when living, or housed, in normal to good conditions (Sorato et al. 2018; Zidar et al. 2018b; Lagisz et al. 2020). When conditions are reduced, or affective state is more directly manipulated to be negative, a shift towards individuals having a more negative, or pessimistic, bias is typically observed (e.g., Lagisz et al. 2020; Neville et al. 2020; Zidar et al. 2018b). Therefore, the ‘optimistic’ behaviour of wild toutouwai towards ambiguous cues in our experiment could also partially reflect the fact that they were living in their natural, enriched environment during testing.

To conclude, here we successfully developed a behavioural test aimed at measuring judgement bias in a wild bird species, based on a paradigm that has previously only been carried out in captive settings. In our wild toutouwai population we observed that birds showed behavioural responses that could be interpreted as them being ‘optimistic’ in their judgement of ambiguous cues. These biased responses varied consistently among individuals over a short timespan, and males tended to approach cues more rapidly than females. We encourage other researchers to confirm these findings in other wild species, and advocate for further experiments to validate judgement bias tests in terms of what inner state (e.g., affective state, food motivation), and cognitive mechanisms may underly observed responses. For research aiming to quantify individual variation in cognitive performance in the wild, our results suggest that we should be mindful of the existence of judgement biases as they can influence responses to experimental set ups. Finally, to improve our understanding of their adaptive nature, future studies should also aim to confirm our observation in other wild species, and to further explore why cognitive biases arise and what consequences they may have for the individual.

## Supplementary Information

Below is the link to the electronic supplementary material.

Supplementary Material 1: The data supporting this paper

Supplementary Material 2: R code for the analyses reported in this paper

Supplementary Material 3: Document containing Tables S1, S2, S3 and Figure S1.

## Data Availability

Data and R code used in this study are available as supplementary material.
