# Supplementary material for: Toutouwai display positive judgement bias when tested in the wild
Source: Anim Cogn. 2025 Oct 29;28(1):84. doi: 10.1007/s10071-025-02004-6 (PMC12572075; doi:10.1007/s10071-025-02004-6)
Supplement: Supplementary file 3 — Supplementary Material 3: Document containing Tables S1, S2, S3 and Figure S1. [file 10071_2025_2004_MOESM3_ESM.docx]

**Supplementary Material supporting the study “*Toutouwai display positive judgement bias when tested in the wild*”**

Authors: Rachael Shaw, Hanne Løvlie

**Table S1**: Descriptive information for the successful test subjects. For the 19 toutouwai that completed all stages of the experiment, the cohort refers to the breeding season in which a toutouwai hatched and age is their approximate age in years during the winter testing period. Also shown is the number of trials required to pass habituation 1, habituation 2 and discrimination training, as well as the rewarded colour and apparatus side the cue was presented on during the discrimination training. See main text for methodological details.

| **Subject** | **Sex** | **Cohort** | **Age (Years)** | **Trials to pass habituation 1** | **Trials to pass habituation 2** | **Trials to pass discrimination** | **Reward colour** | **Reward side** |
| --- | --- | --- | --- | --- | --- | --- | --- | --- |
| Air | F | 2007/2008 | 11.5 | 5 | 5 | 32 | White | Left |
| Apple | F | 2016/2017 | 2.5 | 2 | 4 | 37 | Black | Right |
| April | F | 2013/2014 | 5.5 | 2 | 3 | 14 | White | Left |
| Beebe | F | 2013/2014 | 5.5 | 2 | 3 | 19 | Black | Left |
| Florence | F | 2015/2016 | 3.5 | 2 | 4 | 13 | Black | Right |
| Mayhem | F | 2016/2017 | 2.5 | 2 | 5 | 36 | White | Left |
| McFly | F | 2015/2016 | 3.5 | 2 | 4 | 14 | White | Left |
| Sky | F | 2017/2018 | 1.5 | 2 | 3 | 13 | White | Right |
| Sunny | F | 2016/2017 | 2.5 | 2 | 7 | 40 | Black | Left |
| Ace | M | 2017/2018 | 1.5 | 2 | 3 | 17 | White | Right |
| Champ | M | 2010/2011 | 8.5 | 2 | 3 | 12 | Black | Right |
| Clive | M | 2016/2017 | 2.5 | 2 | 3 | 57 | Black | Left |
| Fred | M | 2017/2018 | 1.5 | 2 | 3 | 37 | Black | Right |
| Jet | M | 2012/2013 | 6.5 | 2 | 3 | 13 | Black | Left |
| Machiavelli | M | 2016/2017 | 2.5 | 2 | 5 | 23 | Black | Right |
| Patrick | M | 2014/2015 | 4.5 | 2 | 3 | 40 | Black | Right |
| Scooby | M | 2012/2013 | 6.5 | 2 | 3 | 12 | White | Left |
| Spark | M | 2014/2015 | 4.5 | 2 | 3 | 48 | Black | Left |
| Tui | M | 2015/2016 | 3.5 | 2 | 5 | 28 | White | Right |
|  |  | **Mean ± SE** | **4.2 ± 0.6** | **2.2 ± 0.2** | **3.8 ± 0.3** | **26.6 ± 3.2** |  |  |

**Table S2:** The order of presentation of each cue type during the cognitive judgement bias test. In total each bird had 24 trials, split across two sessions of 12 trials per day. To counterbalance the order in which birds experienced ambiguous cues, 8 of the birds were given order A, while 11 birds were given order B.

|  | **Trial:** | **1** | **2** | **3** | **4** | **5** | **6** | **7** | **8** | **9** | **10** | **11** | **12** |
| --- | --- | --- | --- | --- | --- | --- | --- | --- | --- | --- | --- | --- | --- |
| **Order A** | **Session 1** | POS | Mid | NEG | Near POS | POS | Near NEG | NEG | Mid | POS | Near POS | NEG | Near NEG |
|  | **Session 2** | NEG | Mid | POS | Near POS | NEG | Near NEG | POS | Mid | NEG | Near POS | POS | Near NEG |
| **Order B** | **Session 1** | POS | Mid | NEG | Near NEG | POS | Near POS | NEG | Mid | POS | Near NEG | NEG | Near POS |
|  | **Session 2** | NEG | Mid | POS | Near NEG | NEG | Near POS | POS | Mid | NEG | Near NEG | POS | Near POS |

**
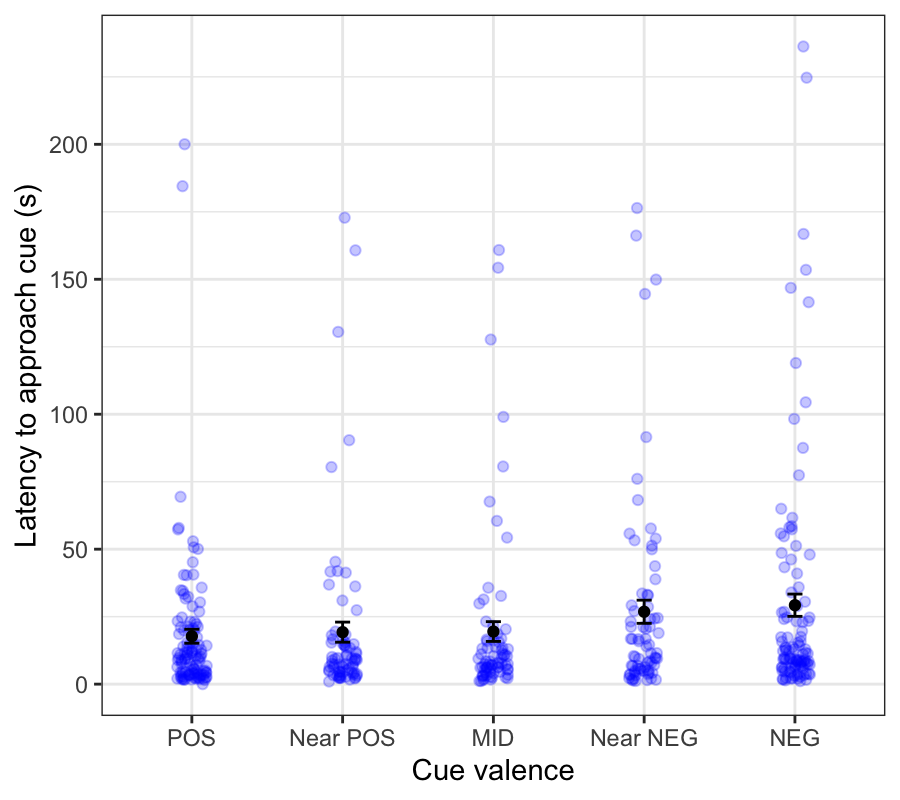
**

**Fig. S1:** Behavioural responses of toutouwai during a judgement bias test. We measured latency (s) to approach each cue type (POS = Positive, Near POS = Near positive, MID = Middle, Near NEG = Near negative, NEG = Negative). Each blue point represents an individual latency measure (points are jittered for visibility). The black points with error bars display the mean approach latency and standard error for each cue type (these are the same means that are shown in Fig. 2 in the main manuscript).

**Table S3:** Post hoc Tukey comparisons of toutouwai behavioural responses toward each cue type in the cognitive judgement bias test. *P* < 0.05 for the comparisons shown in bold.

| **Post hoc comparison** | **Estimate** | **SE** | **Z value** | **P** |
| --- | --- | --- | --- | --- |
| Near POS - POS = 0 | 0.016 | 0.128 | 0.126 | 1.000 |
| MID - POS = 0 | 0.023 | 0.127 | 0.184 | 1.000 |
| Near NEG - POS = 0 | 0.328 | 0.128 | 2.566 | 0.076 |
| **NEG - POS = 0** | **0.395** | **0.115** | **3.432** | **0.005** |
| MID - Near POS = 0 | 0.007 | 0.140 | 0.052 | 1.000 |
| Near NEG - Near POS = 0 | 0.312 | 0.140 | 2.221 | 0.171 |
| **NEG - Near POS = 0** | **0.379** | **0.129** | **2.935** | **0.028** |
| Near NEG - MID = 0 | 0.304 | 0.140 | 2.176 | 0.188 |
| **NEG - MID = 0** | **0.372** | **0.129** | **2.893** | **0.031** |
| NEG - Near NEG = 0 | 0.067 | 0.129 | 0.522 | 0.985 |
